# Supplementary figures and images for: Single-cell ‘omic profiles of human aortic endothelial cells in vitro and human atherosclerotic lesions ex vivo reveal heterogeneity of endothelial subtype and response to activating perturbations
Source: eLife. 2024 Apr 5;12:RP91729. doi: 10.7554/eLife.91729 (PMC10997331; doi:10.7554/eLife.91729)

Annotated blot:

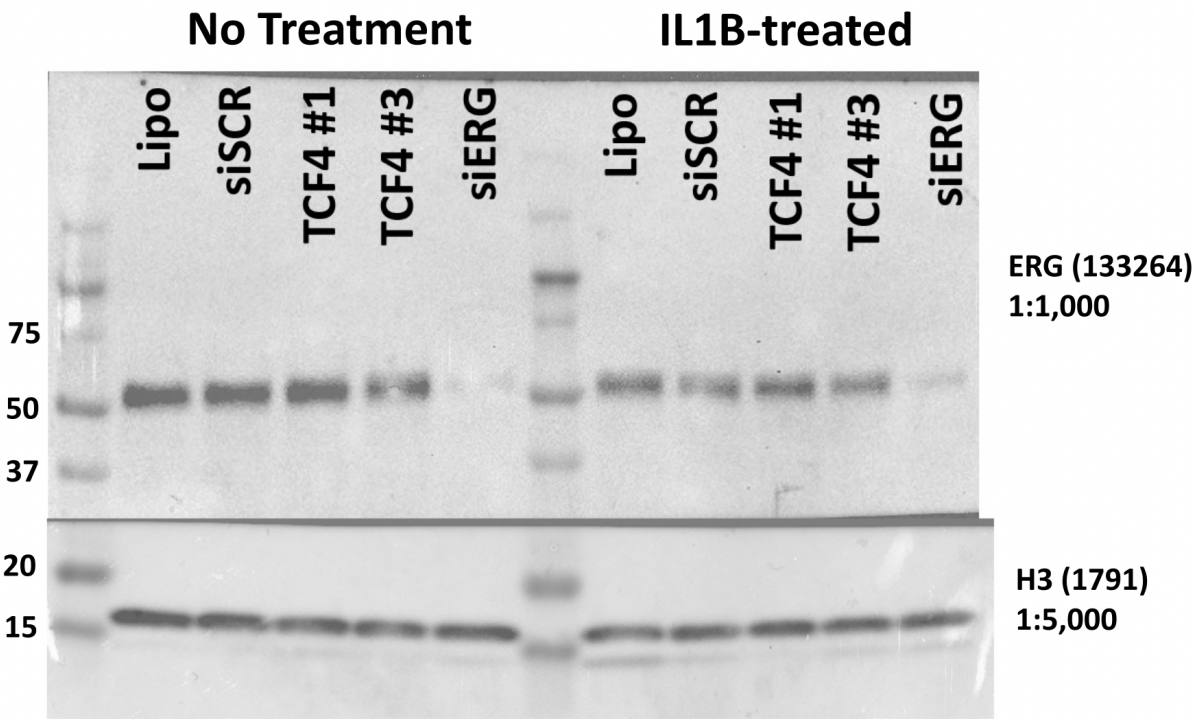

Supplement: Figure 3—source data 1. — The ERG blot (middle) and the H3 blots (bottom) originated from the same gel and membrane, which was cut at about 30 kD, and each piece was blotted for either anti-ERG antibody or anti-H3 antibody. [file elife-91729-fig3-data1.pdf]

No Treatment

IL1B-treated

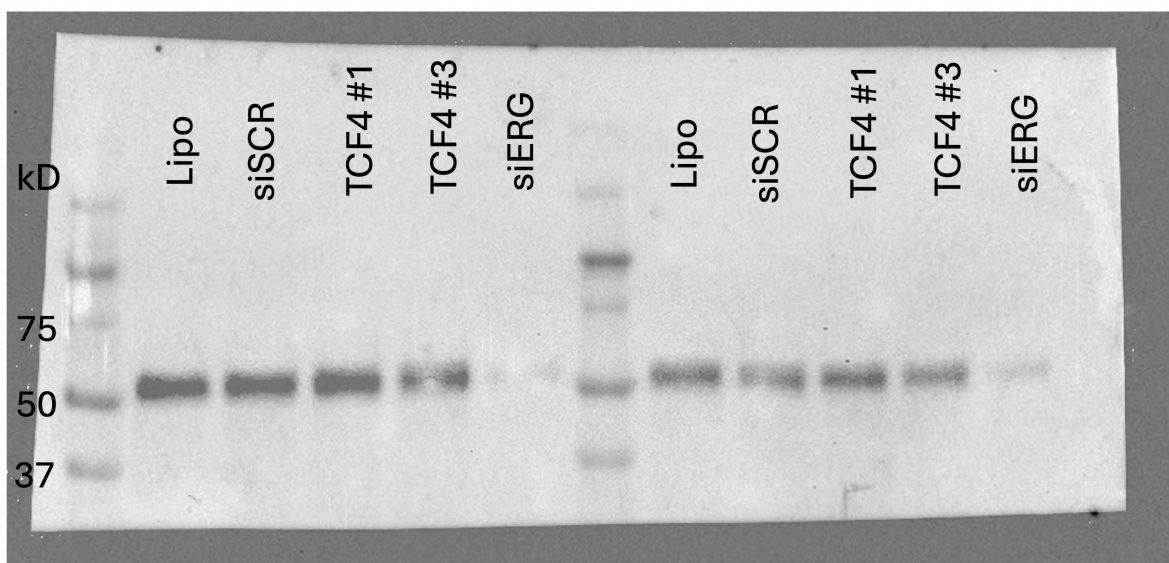

Supplement: Figure 3—source data 2. — Lanes are the same as described in Figure 3—source data 1. [file elife-91729-fig3-data2.pdf]

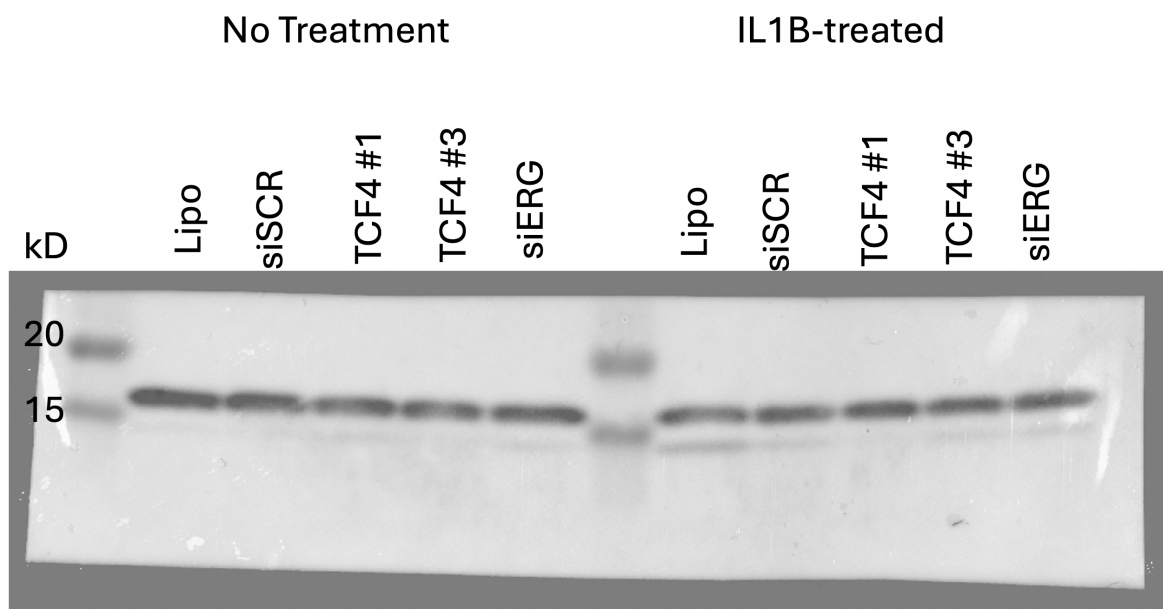

Supplement: Figure 3—source data 3. — Lanes are the same as described in Figure 3—source data 1. [file elife-91729-fig3-data3.pdf]

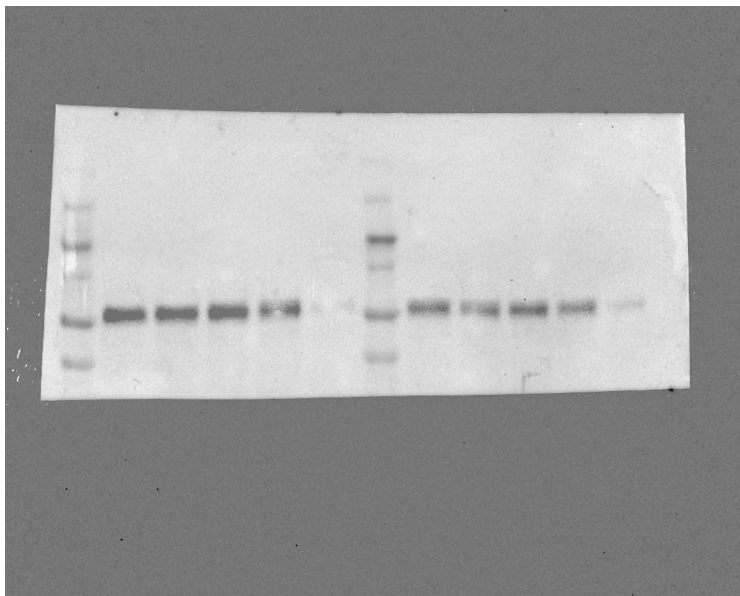

Supplement: Figure 3—source data 4. — Lanes are the same as described in Figure 3—source data 1. [file elife-91729-fig3-data4.pdf]

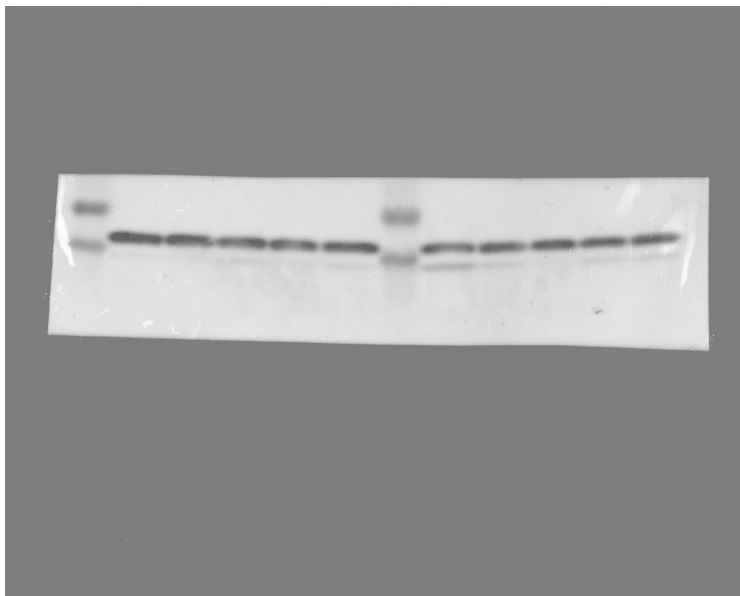

Supplement: Figure 3—source data 5. — Lanes are the same as described in Figure 3—source data 1. [file elife-91729-fig3-data5.pdf]
